# Perceived Severity of COVID-19 and Post-pandemic Consumption Willingness: The Roles of Boredom and Sensation-Seeking

**DOI:** 10.3389/fpsyg.2020.567784

**Published:** 2020-09-16

**Authors:** Shichang Deng, Wangshuai Wang, Peihong Xie, Yifan Chao, Jingru Zhu

**Affiliations:** ^1^School of Management, Shanghai University of International Business and Economics, Shanghai, China; ^2^School of Business, Shanghai University of International Business and Economics, Shanghai, China

**Keywords:** COVID-19, boredom, changes in sensation-seeking expressions, consumption willingness, impulsive buying

## Abstract

The COVID-19 pandemic restricts people’s activities and makes consumer businesses suffered. This study explored the relationship between the perceived severity of COVID-19 and the post-pandemic consumption willingness. Study 1 surveyed 1464 Chinese people in March 2020, found the perceived severity of COVID-19 during the pandemic significantly increased the willingness to consume post-pandemic, and boredom stemming from limited activities and sensation-seeking expressions mediated this effect. Study 2 conducted an experiment with 174 participants in August 2020, found a high level of perceived severity of COVID-19 and the experience of life tedium during the pandemic significantly increased individuals’ impulsive buying tendencies after the pandemic. The results suggested the level of perceived severity of COVID-19 may influence people’s post-pandemic consumption patterns.

## Introduction

No one could have predicted the second decade of the 21st century would begin with a global super pandemic. In just a few months, the novel coronavirus (COVID-19) swallowed more than 3,000 lives and infected more than 80,000 people in China. The Chinese government established unprecedented measures and suspended almost all social activities throughout the country to combat the virus. Although these measures have effectively slowed down the spread of the virus, society has paid a considerable price, especially consumer enterprises. The iResearch Consulting Group (2020) report stated that businesses such as catering, tourism, and transportation were struck during the pandemic due to the order to enforce social distancing, with the net consumer population falling by more than 80%. A column analysis of Beijing Business Daily (2020) also reported the sharp drop in customers from the pandemic led to small and medium-sized retailing and catering enterprises to lose nearly 90% of their income, leaving many businesses in decay. The pandemic hit China’s consumer economy hard in the first quarter of 2020, creating a secondary disaster from COVID-19.

With the pandemic gradually controlled in China, many Chinese businesses have their hopes on a consumption rebound after the pandemic. The Ministry of Commerce of China reported a rebound in consumption in April 2020 (People’s News, 2020). Many business analysts also agree that a spending spree may occur after the pandemic (Beijing Business Daily, 2020; iResearch Consulting Group, 2020). However, what is the psychological reasons for the rise in consumer willingness after the pandemic? Existing research lacks an explanation. This study explores the psychological mechanisms between the perceived severity of COVID-19 and the post-pandemic consumption willingness. We found that during the pandemic, the perceived severity of COVID-19 leads to an increase in boredom state and sensation-seeking expression, which makes the purchasing activity after the pandemic becomes more attractive. We hope this study could provide a reference for similar follow-up researches and consumer enterprises’ post-pandemic business planning.

## Theoretical Background and Hypothesis

### Perceived Severity of COVID-19, Boredom From Limited Activities, and Sensation-Seeking Expressions

The COVID-19 pandemic quickly made headlines in global media after Dr. Zhong Nanshan indicated on the China Central Television (CCTV) News Channel that “it can affirm that this novel coronavirus has human-to-human transmission” on January 20, 2020, and the public soon began to realize the seriousness of the coronavirus. Wuhan city quickly locked down on January 23 after Dr. Zhong’s interview. A week later, all provinces and regions across China launched a first-level public health emergency response (Xinhua News Agency, 2020). Local governments quickly initiated a series of rigorous control methods, such as comprehensive screening and quarantining suspected cases, close monitoring and tracking their contacts, and actively promoting scientific knowledge and expert consensus on coronavirus prevention. However, at the same time, many rumors about the pandemic spread rapidly through online social media, generating a great deal of panic (Wang et al., 2020). Li J. et al. (2020)’s survey of 4,607 Chinese people in February 2020 showed the perceived severity of COVID-19 was as high as 4.09 out of 5 (*SD* = 0.59), which demonstrated that these pandemic-related incidents put people on high alert and led to a dramatic increase in the perceived severity of COVID-19.

The health belief model proposes that perceived severity refers to an individual’s subjective perception of a disease’s serious state, which is influenced by a range of factors related to the current existing reality and anticipation of future events (Green and Murphy, 2014). Weinstein (2000) demonstrated that a high perceived severity of disease causes proactive health-protection behaviors. “Washing hands frequently, wearing masks, not gathering and going out” are the COVID-19 control requirements strongly advocated by the Chinese Government (Xinhua News Agency, 2020). Chinese people actively followed the above pandemic-control instructions when the perceived severity of COVID-19 increased, obeying social distancing rules and locking themselves at home. The survey of Li J. et al. (2020) showed that Chinese people’s social participation levels during the pandemic were as low as 1.75 out of 5 (*SD* = 0.77) since February 2020. Although people’s proactive health-protection behaviors do effectively slowed the spread of coronavirus, the limited activities have also caused a sudden increase in psychological pressure, resulting in different degrees of mental stress (Qiu et al., 2020; Xiang et al., 2020; Zhang et al., 2020).

This study paid particular attention to a psychological consequence of limited activities for a long time: boredom. Boredom is an aversive experience of wanting but not being able to engage in satisfying activities, which occurs when people are unable to focus on desired tasks (Eastwood et al., 2012). Boredom can be seen as a situational state that lacks novel stimuli over a period of time, and low arousal is the most dominant feature of boredom (van Hooft and van Hooff, 2018). The arousal theory states that individuals need a certain amount of external stimulation to maintain the desired activities required by the body; otherwise, they may feel uncomfortable (Reisenzein, 2017). Individuals prefer a moderate level of stimulus; being in a high or low degree of arousal for a long time causes discomfort. A higher level of arousal makes people feel excited, but it also makes them feel nervous, anxious, and irritable. A lower level of arousal makes people feel relaxed; however, it may also cause weariness, depression, and most importantly, boredom (Picard et al., 2016).

A high level of perceived severity of COVID-19 makes most Chinese people exhibit active health-protection behaviors and stay at home, which significantly limits the social activities people can enjoy. Simple and repetitive external stimuli reduce individuals’ arousal levels and create boredom (van Tilburg and Igou, 2017). Long-term activity limitations made people experienced repetitive and monotonous external stimuli; consequently, people’s arousal levels during the pandemic were far below the average (Chao et al., 2020). Although the optimal amount of external stimulation preferred by each individual varies, the long social distancing period has generally caused high levels of boredom in most of the population (Li W. et al., 2020). There were always those who ventured onto the streets and even gathered to play mahjong during the pandemic, despite government calls to reduce going out and gathering (NPR, 2020). The above cases demonstrate that the pandemic restrictions significantly increased people’s boredom.

Long-term boredom states can cause individuals to actively seek out more and stronger complex external stimuli (Reisenzein, 2017). We suggest that the boredom stemming from limited activities during the pandemic leads to an increase in sensation-seeking expression. Sensation-seeking refers to people’s desire for a novel, exciting, and complicated feeling or experience (Zuckerman, 2010). Most researchers conceptualize sensation-seeking as a stable trait (Zuckerman and Aluja, 2015). However, the degree of expression of this trait may be affected by a long period of boredom due to limited activities. Trait activation theory highlights that situational cues may affect how an individual expresses his or her traits (Tett and Burnett, 2003). Lynne-Landsman et al. (2011) found that the social environment influences an individual’s sensation-seeking expression. Lydon-Staley et al. (2020) also showed that individuals express higher than usual sensation-seeking behaviors during the days they consume alcohol, demonstrating that sensation-seeking expression has a within-person variability. Therefore, although the trait of sensation-seeking is relatively stable, one’s expression of sensation-seeking may change depending on the situation. As stated above, elevated perceived severity of COVID-19 led to active health-protective behaviors that made people afraid to leave their homes. Consequently, the monotony of repetitive life from activity limitations reduced people’s arousal levels and increased people’s feelings of boredom (Chao et al., 2020), which resulted in increased sensation-seeking expressions (Dahlen et al., 2004; Jiang et al., 2009; Jee et al., 2010). Those processes let people need more and stronger external stimuli to achieve the desired state of arousal; otherwise, people may feel unpleasant (Zhang et al., 2016). The above mental changes provide a psychological basis for the increased post-pandemic consumption willingness.

### Boredom From Limited Activities, Sensation-Seeking Expressions, and Post-pandemic Consumption Willingness

People’s increased boredom from limited activities and sensation-seeking expressions during the pandemic gave us good reason to speculate that people’s willingness to consume and impulsive buying tendencies may climb significantly after the pandemic is effectively controlled. By satisfying an individual’s needs through payment, consumption is an effective means to elevate arousal levels (Batra and Ghoshal, 2017; Koles et al., 2018). Yan et al. (2016) demonstrated that individuals’ willingness to consume would greatly increase if they try to seek external stimuli to enhance their arousal. Sundström et al. (2019) found that boredom is one of the primary motivators driving people’s buying behavior. Consumers are easily attracted by stimuli, such as advertisements and discounts, when they are bored. Deng and Gao (2015) also showed that sensation-seeking makes individuals actively pursue complex stimuli, so a high level of sensation-seeking expression may result in a significant willingness to consume. As stated above, during the pandemic, a high level of perceived severity of COVID-19 made people reluctant to engage with the outside world (Qiu et al., 2020). The long period of physical and psychological limitations severely deprived people of external stimuli, resulting in increased boredom and sensation-seeking expressions (Chao et al., 2020; Droit-Volet et al., 2020; Kim, 2020). We suggest that after the COVID-19 pandemic is effectively controlled, people are highly likely to engage in a variety of consumption activities precisely because shopping is a complex stimulus that can relieve consumers’ boredom state (Sundström et al., 2019) and satisfy their sensation-seeking needs (Punj, 2011; Deng and Gao, 2015). We hypothesize the following based on the above reasoning:

H1: The perceived severity of COVID-19 during the pandemic will increase the post-pandemic consumption willingness.

H2: The above effect is mediated by boredom from limited activities and sensation-seeking expressions.

Boredom states and sensation-seeking expressions are usually associated with impulse buying because it is a strong psychological stimulus that brings great satisfaction (Dahlen et al., 2004; Iyer et al., 2020). We speculate that since the perceived severity of COVID-19 made an increase in boredom and sensation-seeking expression, it is very likely that the perceived severity of COVID-19 will lead to an elevated impulsivity buying tendency after the pandemic is effectively controlled. The experience of life tedium during the pandemic will play a moderating role in this impact. During the quarantine, many people were restless because of the tedium of life, but many people also found new pleasures, such as cooking or learning a new musical instrument (Droit-Volet et al., 2020). Due to the experience of life tedium greatly improves one’s boredom states and sensation-seeking expressions, we suggest that the impulse buying tendency after the pandemic may decrease if an individual’s prolonged homestay was filled with new things. Conversely, an individual’s tendency to impulsively buying after the pandemic may significantly increase if he or she felt life was tedious during a long period of quarantine. We hypothesized the following based on the above reasoning:

H3: High levels of perceived severity of COVID-19 and experience of life tedium during the pandemic significantly increased individuals’ impulse buying tendency after the pandemic.

We tested the three above hypotheses through two studies. A questionnaire modeling tested H1 and H2, which provided an aggregate survey of the relationship between perceived severity of COVID-19 and post-pandemic consumption willingness, as well as the mediators between them. A behavioral experiment tested H3, which provided evidence of how the perceived severity of COVID-19 and the experience of life tedium during the pandemic affected one’s impulse buying tendency after the pandemic.

## Study 1

Study 1 aims to use the questionnaire modeling method to test H1 and H2 (i.e., whether perceived severity of COVID-19 increased ones’ post-pandemic consumption willingness through the mediating roles of boredom from limited activities and sensation-seeking expressions). We conducted this study in March 2020. At this time, the number of new COVID-19 cases in China has been gradually decreasing, but the overall situation of the pandemic is still serious.

### Procedure and Participants

We posted a set of questionnaires on a Chinese web-based survey platform on March 15, 2020. Within 3 days, 1464 people responded in full for a small cash reward. The participants (665 females, *M*age = 28.40, *SD* = 6.84) came from all regions in China. Among them, 247 were students (16.90%), 1079 had formal jobs (73.70%), 34 had part-time jobs (2.30%), 92 were freelance (6.30%), and 12 were unemployed (0.80%).

### Measures

We asked participants to respond to the questionnaires in the following order (see Supplementary Material for full items).

#### Perceived Severity of COVID-19

Referring to the “COVID-19 Pandemic Perception Questionnaire (2nd round),” published by the Sun Yat-sen University team (2020), 6 items suitable for the topic of this study were selected after authors’ discussion (e.g., “I often suspect that people around me may be infected by the coronavirus.”) Participants responded on a 5-point Likert scale from 1 (very much disagree) to 5 (very much agree), with higher scores indicating a *higher* level of perceived severity of COVID-19. In this study, the 6 items have a good unidimensional structural validity (goodness-of-fit of CFA: χ^2^ = 50.58, *df* = 9, RMSEA = 0.06, SRMR = 0.02, CFI = 0.98, TLI = 0.97), with factor loading between 0.74 and 0.48 and the Cronbach’s α is 0.81.

#### Boredom From Limited Activities

We adopted the low arousal subscale of the Chinese Multidimensional State Boredom Scale (CMSBS), which was developed by Liu et al. (2013). The CMSBS contains five subscales: inattentiveness, perceived slowing of time, low arousal, high arousal, and a desire to engage in more exciting activities. Of these, the low arousal state best suits this study because compared with the other four subscales, it best described a low mental arousal state. This subscale consists of 5 items. The phrase “during the period of home staying” was added to each item, for example, “during the period of home staying, everything is repetitive and boring for me because of the restrictions on my activities.” Participants responded on a 5-point Likert scale from 1 (very much disagree) to 5 (very much agree), with higher scores indicating a *higher* boredom state during the pandemic. In this study, the Cronbach’s α of this subscale is 0.86.

#### Sensation-Seeking Expression

Several instruments have been developed for different research purposes for assessing sensation-seeking. The 40-item Sensation-Seeking Scale Form V (SSS-V) is the most widely used among these instruments (Zuckerman and Aluja, 2015). However, large-scale surveys require a shorter measurement tool, and sensation-seeking expression closely relates to an individual’s culture (Wang et al., 2000; Agrusa et al., 2007). Therefore, we adopted the Chinese Brief Sensation-seeking Scale in this study, which Hoyle et al. (2002) derived from the SSS-V and Chen et al. (2013) culturally adapted. This scale consists of 8 items and mainly measures the behavioral tendencies of sensation-seeking individuals. The phrase “during the pandemic” was added before each item to evaluate participants’ sensation-seeking expressions during that period, for example, “during the pandemic, I always liked to do things that I had not done before.” Participants responded on a 5-point Likert scale from 1 (very much disagree) to 5 (very much agree), with higher scores indicating a *higher* sensation-seeking tendency during the pandemic. The Cronbach’s α of this scale was 0.76 in this study.

#### Post-pandemic Consumption Willingness

Six items were developed to measure this variable based on the general psychometric procedure, i.e., when the pandemic is over, “…I want to go out and eat some delicious food”, “…I want to have more shopping and buying”, “…I will compensate for my pent-up spend desire and satisfy myself by buying more things”, “…my consumption desire will increase significantly than before the pandemic”, “…I want to buy something that I haven’t bought before”, and“…I will spend more and have fun in time.” Participants responded on a 5-point Likert scale from 1 (very much disagree) to 5 (very much agree), with higher scores indicating a *higher* post-pandemic consumption willingness. The 6 items have a good unidimensional structural validity (goodness-of-fit of Confirmatory Factor Analysis: χ^2^ = 24.92, *df* = 9, RMSEA = 0.04, SRMR = 0.02, CFI = 0.99, TLI = 0.99) in this study, with factor loading between 0.79 and 0.39 and the Cronbach’s α is 0.82.

#### Control Variables

Considering the pandemic affected many people’s financial income, which is a significant consumption-related factor, this study also asks the question “has the pandemic affected your economic income?” Participants answered on a 5-point Likert scale from 1 (no impact at all) to 5 (the impact is huge). Furthermore, considering that life satisfaction during the pandemic may also affect the post-pandemic consumption willingness, this study adopted a single-item scale developed by Cheung and Lucas (2014) (i.e., “in general, are you satisfied with your life situation during the pandemic?”) Participants responded on a 5-point Likert scale from 1 (very much disagree) to 5 (very much agree).

### Results

First, we examined the differences of post-pandemic consumption willingness between demographic variables. An independent *t*-test found the score of females on post-pandemic consumption willingness (*M*_*females*_ = 3.89, *SD* = 0.76) was slightly higher than that of males (*M*_*males*_ = 3.81, *SD* = 0.79), but the difference was not significant [*t*(1462) = 1.90, *p* = 0.057, Cohen’s *d* = 0.10]. The correlation between age and post-pandemic consumption willingness also failed to reach a significant level (*r* = −0.05, *p* = 0.052). Those results demonstrated that the post-pandemic consumption willingness is a general trend, with little change in demographics.

Next, Table 1 shows the Pearson correlations between variables. A significant positive correlation can be found between the perceived severity of COVID-19 and post-pandemic consumption willingness (*r* = 0.29, *p* < 0.001). Furthermore, there were also significant positive correlations between boredom from limited activities (*r* = 0.26, *p* < 0.001) and sensation-seeking expressions (*r* = 0.37, *p* < 0.001) regarding the post-pandemic consumption willingness.

**TABLE 1:** Pearson correlations between variables (*N* = 1464).

	***M***	***SD***	**1**	**2**	**3**	**4**	**5**
1. Perceived severity of COVID-19	3.33	0.87	–				
2. Boredom from limited activities	3.19	0.96	0.53***	–			
3. Sensation-seeking expressions	3.10	0.71	0.43***	0.52***	–		
4. Post-pandemic consumption willingness	3.84	0.78	0.29***	0.26***	0.37***	–	
5. Impact of the pandemic on income	3.62	1.14	0.31***	0.27***	0.24***	0.10***	–
6. Life satisfaction during the pandemic	3.23	0.99	−0.12***	−0.20***	0.03	0.05	−0.04

***p < 0.001.

In addition, the impact of the pandemic on income significantly and positively correlated with the post-pandemic consumption willingness, but the effect size was at a low level (*r* = 0.10, *p* < 0.001). Life satisfaction during the pandemic did not significantly correlate with the post-pandemic consumption willingness (*r* = 0.05, *p* = 0.059). The results suggested little relationship exists between the two control variables and the dependent variable.

We used a structural equation model to further test H1 and H2 based on Hayes’ (2013) Model 6. Our model contained both observed and latent variables and was computed with 2000 bootstrapping through Maximum-Likelihood Estimation. The model’s goodness-of-fit was acceptable (χ^2^ = 1850.32, *df* = 265, RMSEA = 0.06, SRMR = 0.08, CFI = 0.89, TLI = 0.87). The results indicated the perceived severity of COVID-19 had led to a significant increase in boredom from limited activities (β = 0.63, *p* < 0.001), which then result in a significant rise in sensation-seeking expressions (β = 0.31, *p* < 0.001), and eventually made a significantly elevation in post-pandemic consumption willingness (β = 0.33, *p* < 0.001).

**FIGURE 1:**
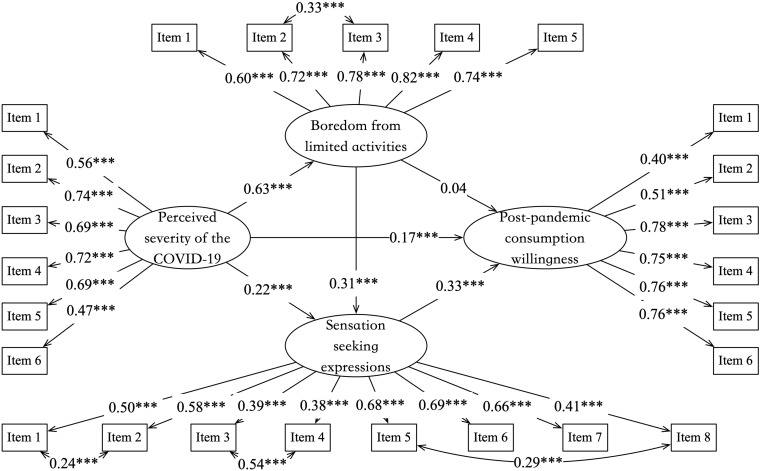
Perceived severity of COVID-19 results in an increased post-pandemic consumption willingness through the mediating roles of boredom from limited activities and sensation-seeking expressions (Study 1). Numbers are the standardized path coefficient, ***p < 0.001.

Figure 1 also shown that the effect of boredom from limited activities on post-pandemic consumption willingness was not significant (β = 0.04, *p* = 0.402), as well as the indirect effect through boredom only (β = 0.02, *p* = 0.404). Therefore, the indirect effects of the perceived severity of COVID-19 on post-pandemic consumption willingness were realized through sensation-seeking expressions only (β = 0.07, *p* < 0.001), and boredom and sensation-seeking expressions in succession (β = 0.06, *p* < 0.001). The total indirect effects (β = 0.16, *p* < 0.001) account for almost half of the total effects (β = 0.33, *p* < 0.001).

### Discussion

Study 1 supports H1 and H2. It shows a general trend that the perceived severity of COVID-19 could lead to increased boredom from limited activities, then result in heightened sensation-seeking expressions. As a consequence, these changes led to a raised post-pandemic consumption willingness. The results of Study 1 indicate that in March 2020, in which the pandemic in China was still severe, the perceived severity of COVID-19 was closely related with a climbed post-pandemic consumption willingness. Boredom and sensation-seeking expressions are often associated with impulsive consumption (Dahlen et al., 2004; Sundström et al., 2019), so does the perceived severity of COVID-19 makes people more likely to consume impulsively after the pandemic? We examined this speculation in Study 2.

## Study 2

Study 2 aims to replicate and extend the findings of Study 1. We examined whether the perceived severity of COVID-19 and the experience of life tedium during the pandemic elevated people’s impulsive buying tendencies after the pandemic was effectively controlled by manipulating these two variables (i.e., test H2). We conducted a behavioral experiment in August 2020. At this time, the pandemic has been brought under control in most parts of China, with only a few sporadic new cases.

### Participants and Procedure

Participants (174 people, 74 females, *M*age = 28.06, *SD* = 6.78) from a Chinese web-based survey platform were randomly assigned to a 2 (perceived severity of COVID-19: severe vs. not severe) × 2 (experience of life tedium: tedious vs. not tedious) between-subjects design from August 4–6, 2020. In the manipulation of the perceived severity of COVID-19, the severe group watched a 90-s news video that emphasizing the virus was still serious in China. The not-severe group watched a similar length video; however, that video stating the COVID-19 pandemic was effectively controlled in China. Both news videos were clipped from authoritative Chinese media outlets (see Supplementary Material). In the manipulation of the experience of life tedium during the pandemic, the tedious group was asked to describe in detail “how your life was repetitive and tedious during the long period of home staying.” The not-tedious group was asked “how your life was full of new things during the long period of home staying.” Next, all participants were required to respond to the following items from 1 (very much disagree) to 5 (very much agree).

#### Items for Manipulation Check

Three items from Xin (2020) were adopted to measure participants’ perceived severity of COVID-19 (e.g., “I feel that if I am not careful, my family or I am very likely to infected by the coronavirus,” “I feel that the current pandemic situation is very serious,” and “I feel that it is tough to control the pandemic effectively.”) The Cronbach’s α of those three items is 0.78. Two items from Study 1 were used to measure participants’ experience of life tedium during the pandemic (one from the Boredom Scale: “during the period of home staying, everything is repetitive and boring for me because of the restrictions on my activities” and one from the Sensation-seeking Scale: “during the period of home staying, I would do anything as long as it exciting and stimulating.”) The Cronbach’s α of those two items is 0.86.

#### Items for Impulsive Buying Tendencies After the Pandemic

Participants were first asked to read the following text: “Now, except for a few regions, the pandemic in China has been effectively controlled. In your community, several large shopping malls are planning a large-scale shopping festival, and they will cater to all aspects of the consumer needs such as household goods, entertainment, leisure, sports, and many more.” Then, participants were required to respond to the following five items revised from Sharma et al. (2014): In this shopping festival, I “…will not think too much before buying what I like”; “…will buy things if I like it”; “…will tempted to choose what I like”; “…will not think too much about the consequences of choosing what I like”; and “…will chose what I like as quickly as possible, before I change my mind.” The Cronbach’s α of those items is 0.80.

### Results

Independent *t*-tests showed the manipulation of the perceived severity of COVID-19 [*M*_*severe (not severe)*_ = 3.43(2.38), *SD*_*severe (not severe)*_ = 0.91(0.72), *t*(172) = 8.49, *p* < 0.001, Cohen’s *d* = 1.29] and the experience of life tedium during the pandemic [*M*_*tedious (not tedious)*_ = 3.98(2.90), *SD*_*tedious (not tedious)*_ = 0.77(1.14), *t*(172) = 7.33, *p* < 0.001, Cohen’s *d* = 1.11] were both effective. A 2 × 2 ANOVA on impulsive buying tendencies after the pandemic revealed two significant main effects [perceived severity of COVID-19: *F*(1, 170) = 34.39, *p* < 0.001, η^2^*_*p*_* = 0.17; experience of life tedium during the pandemic: *F*(1, 170) = 14.08, *p* < 0.001, η^2^*_*p*_* = 0.08] and a significant interaction [*F*(1, 170) = 10.34, *p* = 0.002, η^2^*_*p*_* = 0.06].

**FIGURE 2:**
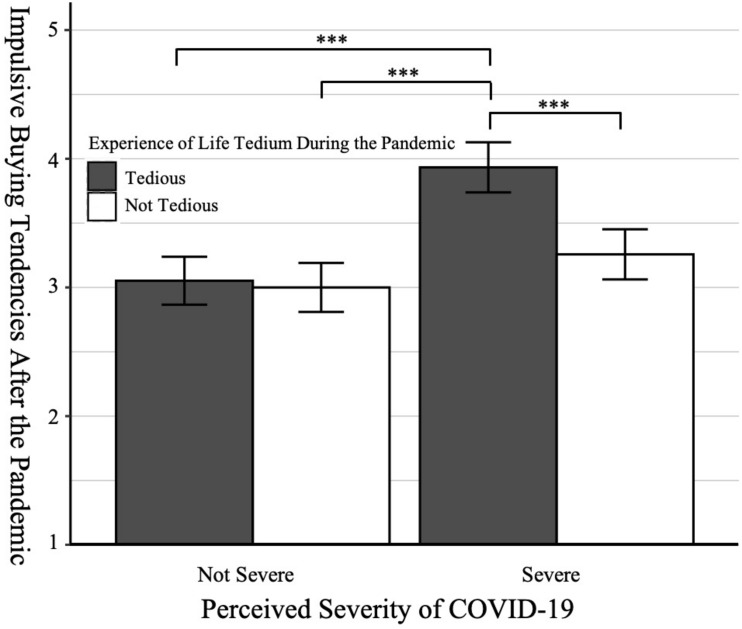
Participants’ impulsive buying tendencies after the pandemic across different conditions (Study 2). Error bars are 95% confidence intervals.

Post Hoc tests found that participants’ post-pandemic impulsive buying tendencies was the highest in the condition of high perceived severity of COVID-19 and high experience of life tedium during the pandemic (*M*_*severe and tedious*_ = 3.93, *SD* = 0.50), which was significantly higher than the condition of high perceived severity and low experience of life tedium [*M*_*severe and not tedious*_ = 3.26, *SD* = 0.81, *t*(170) = 4.84, *p*_*tukey*_ < 0.001, Cohen’s *d* = 1.06], the condition of low perceived severity and high experience of life tedium [*M*_*not severe and tedious*_ = 3.05, *SD* = 0.58, *t*(170) = 6.45, *p*_*tukey*_ < 0.001, Cohen’s *d* = 1.38], and the condition of low perceived severity and low experience of life tedium [*M*_*not severe and not tedious*_ = 3.00, *SD* = 0.58, *t*(170) = 6.76, *p*_*tukey*_ < 0.001, Cohen’s *d* = 1.46]. See Figure 2.

### Discussion

The results of Study 2 support H2. The perceived severity of COVID-19 and the experience of life tedium during the pandemic jointly influenced people’s impulsive buying tendencies after the pandemic. It indicates that in August 2020, in which the pandemic in China was basically controlled, people are more likely to satisfy their stimulus-seeking needs through impulse consumption if they are at high levels of both variables.

## General Discussion

### COVID-19 and Post-pandemic Consumption Willingness

Studies have shown that whether in China (Yuan et al., 2020), the United Kingdom (Chronopoulos et al., 2020), Scandinavia countries (Andersen et al., 2020), or the United States (Cox et al., 2020), the COVID-19 pandemic limited consumers’ activity and led to a significant decline in spending. Our findings suggest this phenomenon may change after the pandemic is adequately controlled. Based on the survey results of 1464 people (Study 1) in March 2020, we see that individuals’ post-pandemic consumption willingness is relatively high (3.84 out of 5, *SD* = 0.78), which implies people’s spending may bounce back after the pandemic.

We suggest the psychological basis for this potential post-pandemic consumption rebound is that individuals are motivated to seek external stimuli to relieve the boredom stemmed from limited activities and to satisfy their sensation-seeking needs. The arousal theory demonstrates that simple and repetitive stimuli reduce individuals’ arousal levels. In the long run, people may actively seek out more significant and complicated external stimuli to restore their desired arousal level. During the pandemic, a high perceived level of severity of COVID-19 led people to be afraid of contact with the outside world, resulting in minimal activities that individuals could participate in. Low-level stimulation for months made people more likely to feel bored, anxious, and irritable (Chao et al., 2020; Li J. et al., 2020; Xiang et al., 2020; Zhang et al., 2020). Consumption is an activity that can quickly lead to novel stimuli. Di Muro and Murray (2012) found that consumers experiencing negative emotions prefer to choose goods that are inconsistent with their current arousal level. Those consumers attempt to escape their emotional discomfort and find their preferred optimal arousal level through consumption. The results of Study 1 demonstrate the high levels of boredom from limited activities and sensation-seeking expressions have a strong positive effect on people’s post-pandemic consumption willingness. During the International Workers’ Day holiday (May 1) in 2020, China saw a significant rebound in tourism numbers (Financial Times, 2020), which suggests people are very likely to meet their demand for external stimulus through consumption.

Our findings echoed other independent studies. Based on samples from the United States, Kim (2020) found the perceived threat of COVID-19 has a close relationship with variety-seeking because the pandemic limited individuals’ activity, therefore people display a high motivation to increase freedom and restore control. It suggests that the impact of the perceived severity of COVID-19 is cross-cultural.

### COVID-19 and Impulsive Buying Tendencies After the Pandemic

Study 1 confirms the perceived severity of COVID-19 is strongly associated with increased boredom and sensation-seeking expressions during the pandemic, which is often closely related to impulsive buying behaviors (Dahlen et al., 2004; Deng and Gao, 2015; Sundström et al., 2019). Study 2 found significant main and interaction effects of both the perceived severity of COVID-19 and the experience of life tedium during the pandemic on impulse buying tendencies. Individuals are highly likely to exhibit an impulsive buying tendency in cases when both of the above variables are at a high level. Study 2 echoes the findings of Li M. et al. (2020), which states impulsive consumption is a typical behavior people often present during public health emergencies. Moreover, Li M. et al. (2020) found the pandemic’s severity positively affects people’s impulsive consumption, and individuals’ perceived control and materialism mediate this effect. Our study complements another path of this effect, that is, perceived severity of COVID-19 and experience of life tedium during the pandemic can also lead to an increased impulsive buying tendency. It demonstrated that the perceived severity of COVID-19 might affect not only the willingness to consume after the pandemic, but also people’s consumption patterns in the future.

Extending the findings of Study 2, we speculate that in addition to impulsive buying tendencies, the perceived severity of COVID-19 and experience of life tedium during the pandemic may also increase a variety of impulsive behaviors. van Rooij et al. (2020) found that in the United States, impulsivity during the pandemic led to a violation in coronavirus control measures. Mesa Vieira et al. (2020) also found that a sharp rise in the divorce rate in China during the pandemic may be associated with increased impulsive decisions. The results of Study 2 suggest that lowering the perceived severity of COVID-19 and experience of life tedium during the pandemic could alleviate people’s impulsivity, thereby reducing the likelihood of making poor decisions.

### Practical Implications and Directions for Further Research

For consumer businesses, it is important to not only prepare for the rapid rebound in consumption after the pandemic, but also to prepare a plan for the normalization of consumption after the rebound weakens. In other words, consumer enterprises must understand that the rebound in consumption will not stem from a sudden increase in society’s spending power, but from the urgent need for consumers to relieve their boredom from limited activities and satisfy sensation-seeking needs. Therefore, consumer enterprises should conduct more forward-looking marketing research and understand consumers’ psychological changes to make the right decisions.

We also advocate that consumers be rational in their purchasing after the pandemic and beware of impulsive buying decisions and overconsumption. On the one hand, after long-term low levels of arousal, moderate consumption could help people restore their perceptual stimulation to their ideal arousal levels. On the other hand, excessive consumption may lead to negative results, such as excessive debt and resource waste (Deng and Gao, 2015; Lee and Ahn, 2016).

Future research should pay attention to the differences in consumption willingness between regions. In China, the COVID-19 outbreak was centered in Wuhan City, Hubei Province. People in the epicenter of the pandemic experienced stricter control measures and had a much higher perceived severity of COVID-19 (Dai et al., 2020). The Yerkes-Dodson law states that either too high or too low levels of psychological stimulation are not conducive to achieving the best mental state. Wuhan City lifted its lockdown on April 8, 2020. After 76 scary days and nights, will the spending spree of those in the epicenter be more vigorous, or will it be business as usual? It is subject to follow-up observation.

Future studies should also focus on the pandemic’s long-term impact on consumer behavior. The COVID-19 pandemic caused long-term, continuous, high-intensity, and traumatic group psychological stress to the people of China and to the world. It could change many people’s views of consumption, making some consumer industries decline while others rise. What new consumption drivers will form by this profound collective memory of a generation? This question is beyond the scope of this study and is left for subsequent studies to explore.

### Limitations

There are three limitations to this study. First, this study lacks distinctions between different consumption types. The pandemic impacted human connection, leaving a significant portion of the population apprehensive about socializing. Therefore, the consumption scenario is better further subdivided into socially based consumption (e.g., bar parties) and non-socially based consumption (e.g., traveling alone), because of the psychological basis of these consumption activities is different.

Second, selecting a subscale may not be a good choice for evaluating low arousal states of boredom. These measurements constitute various dimensions, in addition to being highly variable depending on the time of day the individual responds (Adan and Guàrdia, 1993). Therefore, a multidimensional measure approach should be incorporated to measure low arousal states of boredom to assess the fine effect of the perceived severity of COVID-19 on this variable.

Third, both the arousal state and sensation-seeking closely relate to the individual difference in circadian typology, which associates with various psychological symptoms (Prat and Adan, 2013). Therefore, circadian typology may determine mediation. This study only used questionnaires at a rough level to investigate people’s overall levels of boredom. Follow-up studies should fully consider the circadian changes of an individual’s activation to obtain more accurate results.

## Data Availability Statement

The datasets generated during and/or analyzed during the current study are available from the corresponding author on reasonable request.

## Ethics Statement

The studies involving human participants were reviewed and approved by the research ethics board of Shanghai University of International Business and Economics. The patients/participants provided their written informed consent to participate in this study.

## Author Contributions

SD and WW devised the project, the main conceptual ideas, and proof outline. PX worked out most of the technical details and performed the numerical calculations. JZ collected the research data. SD, WW, and YC wrote the manuscript. All authors contributed to the article and approved the submitted version.

## Conflict of Interest

The authors declare that the research was conducted in the absence of any commercial or financial relationships that could be construed as a potential conflict of interest.
