# Supplementary material for: Perceived Severity of COVID-19 and Post-pandemic Consumption Willingness: The Roles of Boredom and Sensation-Seeking
Source: Front Psychol. 2020 Sep 16;11:567784. doi: 10.3389/fpsyg.2020.567784 (PMC7525206; doi:10.3389/fpsyg.2020.567784)
Supplement: Supplementary file 1 [file Data_Sheet_1.docx]

Supplementary Material

# Items of the questionnaires used in Study 1

The items are actually used in Chinese. The following English translation is only for readers to understand the research contents.

The following items are all responded on a 5-point Likert scale from 1 (not at all) to 5 (very much in line)

| *Perceived severity of COVID-19*.   1. I am worried that my family and I will be infected with the new coronavirus. 2. During the pandemic, I sometimes thought, "will there be a virus on this thing?" 3. I believe the pandemic will breakout again. 4. Things around me always remind that the pandemic is never far away. 5. I often suspect that people around me may be infected by the coronavirus. 6. I will avoid contact with others, believing that contact increases the risk of infection. |
| --- |
| *Boredom from limited activities.*   1. During the pandemic, I was bored by restrictions on movement. 2. During the pandemic, I was frustrated by restrictions on movement. 3. During the pandemic, I felt empty because of restrictions on movement. 4. During the pandemic, everything was monotonous and repetitive and boring for me because of the restrictions on my activities |
| *Sensation seeking expressions*   1. During the pandemic, I was interested in almost every new thing. 2. During the pandemic, I always liked to do things I had not done before 3. During the pandemic, I would feel uncomfortable for staying in the same place for too long. 4. During the pandemic, if I do the same thing for a long time, I will be restless. 5. During the pandemic, I prefer to interact with friends in a variety of ways. 6. During the pandemic, new stimulation always makes me very happy. 7. During the pandemic, I was willing to do anything as long as it was exciting and stimulating. 8. During the pandemic, in order to alleviate boredom, I am willing to take some risks. |
| *Post-pandemic consumption willingness*   1. When the pandemic is over, I want to go out and eat some delicious food. 2. When the pandemic is over, I want to have more shopping and buying. 3. When the pandemic is over, I will compensate for my pent-up spend desire and satisfy myself by buying more things. 4. When the pandemic is over, my consumption desire will increase significantly than before the pandemic 5. When the pandemic is over, I want to buy something that I haven’t bought before. 6. When the pandemic is over, I will spend more and have fun in time. |

#
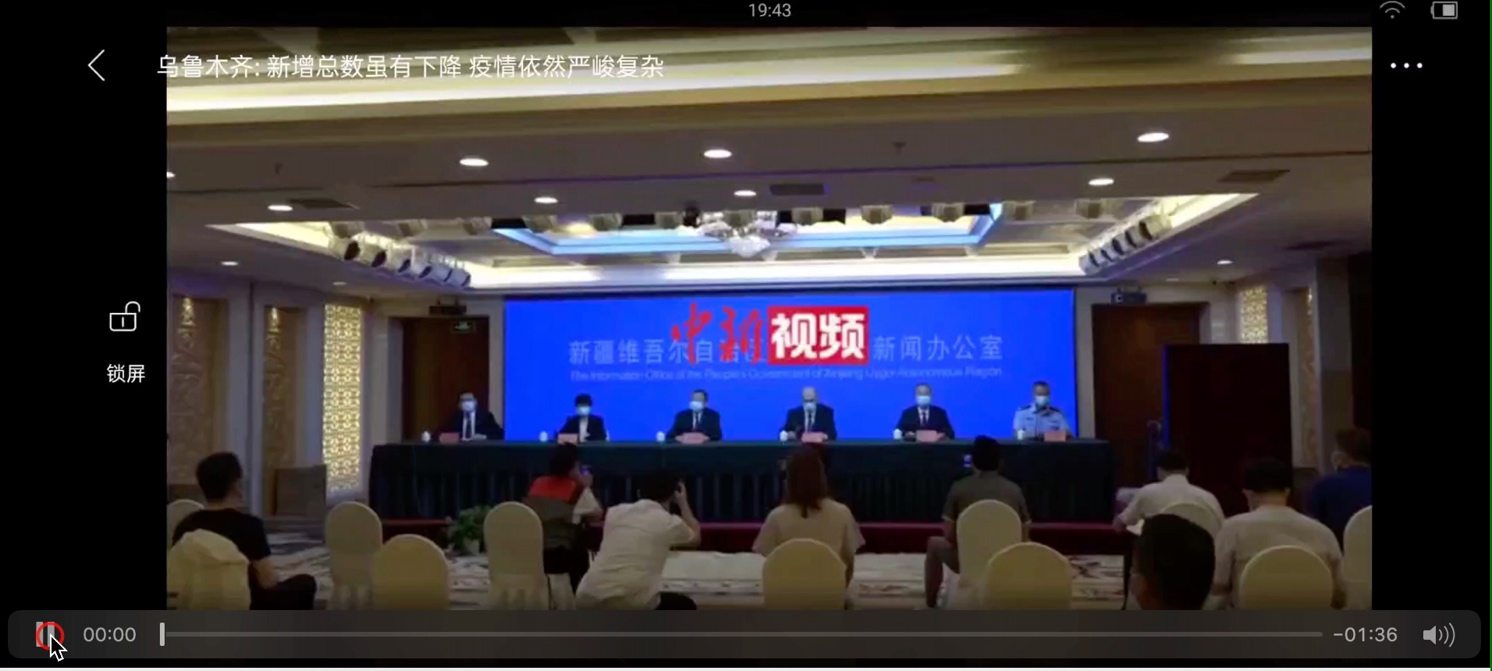
Videos for manipulating the perceived severity of COVID-19

**News video about the COVID-19 pandemic had already effectively controlled in China**


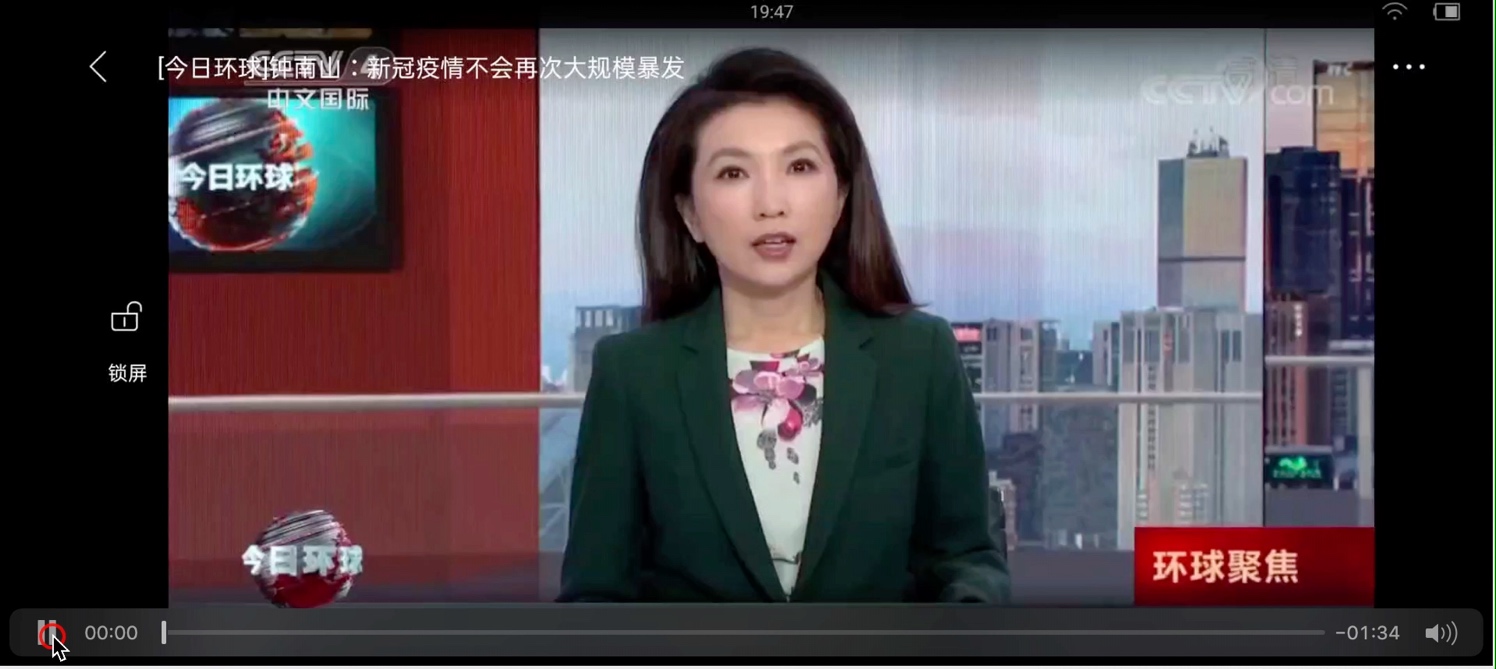
Video title: 钟南山：新冠疫情不会再次大规模暴发 (Dr. Zhong Nanshan: COVID-19 pandemic will not break out again on a large scale)

**News video about the COVID-19 pandemic is still severe in China**

Video title: 乌鲁木齐：新增总数虽有下降，疫情依然严峻复杂 (Urumqi: although the total number of new increases in COVID-19 infection has declined, the pandemic situation is still grim and complicated)
